# Recognition of others’ interoceptive states in those with and without eating disorders

**DOI:** 10.1186/s12888-024-05615-4

**Published:** 2024-02-28

**Authors:** Chandni Gajperia, Jennifer McBride, Janet Treasure, Valentina Cardi, Rebecca Brewer

**Affiliations:** 1 Royal Holloway, University of London, Egham Hill, Egham, London, TW20 0EX UK; 2 King’s College London, London, UK; 3 University of Padova, Padova, Italy

**Keywords:** Interoception, Social perception, Alexithymia, Eating disorders, Anorexia nervosa

## Abstract

**Background:**

The ability to recognize one’s own emotions is associated with one’s ability to recognize others’ emotions. Beyond the domain of emotion, however, the relationship between recognition of one’s own internal states (interoception) and others’ interoceptive states has not been investigated, either in the typical population or clinical groups with interoceptive difficulties (e.g. eating disorders; EDs).

**Method:**

This study investigated recognition of one’s own and others’ internal states in adults with and without eating disorders, using a high frequency visual noise paradigm. Participants completed self-report measures of interoception, alexithymia (difficulties recognising one’s own emotional internal states) and ED symptomatology, and the Heartbeat Counting Task measure of cardiac interoceptive accuracy.

**Results:**

Alexithymia was significantly negatively correlated with recognition of others’ interoceptive states. EDs were not associated with difficulties recognising others’ interoceptive states.

**Conclusions:**

The ability to recognise one’s own emotional internal states is associated with the recognition of others’ interoceptive states, which may contribute to social skills and the ability to care for others.

**Supplementary Information:**

The online version contains supplementary material available at 10.1186/s12888-024-05615-4.

## Background

Interoception refers to the perception of the body’s internal signals, such as cardiac, respiratory and gastric signals, as well as temperature, itch, nausea, pain, and muscle fatigue, although multiple variations of this definition have been proposed [1]. Recent models of interoception distinguish between different domains of interoception, in particular interoceptive attention (the extent to which interoceptive signals are the object of one’s attention) and interoceptive accuracy (one’s ability to perceive internal signals correctly) [2–4]. There are substantial individual differences in interoception, with atypical interoceptive accuracy and attention being implicated in multiple clinical conditions [5, 6], such as Autism Spectrum Disorder [7–10], Attention-Deficit/Hyperactivity Disorder [11], Obsessive Compulsive Disorder [12, 13], schizophrenia [14], alcohol and substance abuse [15–17], depression [18–22], anxiety and panic disorder [22–24], and Eating Disorders (EDs) [25, 26]. While the ability to perceive and interpret *one’s own* interoceptive states, particularly in clinical conditions, has been the focus of much research, very little work has focused on the ability to recognise *others’* interoceptive states. The current study investigated the ability to recognise others’ interoceptive states, and its relationship with perception of one’s own internal signals, in the typical population and a clinical group where deficits in interoceptive accuracy and attention are common (those with EDs). The relationship between recognition of one’s own and others’ interoceptive states is of interest in the general population, as well as in all clinical groups characterised by interoceptive atypicality, as if a relationship is observed, training to improve one’s own interoception might lead to improved recognition of others’ interoceptive states, and in turn more successful social interactions and relationships. As an initial research step, EDs were investigated in the current study because i) they are arguably the condition most intuitively associated with interoceptive difficulties, and ii) individuals with EDs appear to experience difficulties with emotion recognition, social interactions, and relationships [27].

EDs, such as Anorexia Nervosa (AN), Bulimia Nervosa (BN) and Binge Eating Disorder (BED)^1^ are characterized by atypical and inappropriate consumption of food, including restricted food intake, bingeing, and purging behaviors [35]. It is unsurprising that individuals with EDs often exhibit atypical interoception, as difficulties perceiving or attending to hunger and satiety (both interoceptive signals) may reduce one’s ability to eat according to physiological requirements (intuitive eating), as observed in EDs [36]. Indeed, intuitive eating and body mass index (BMI) are positively associated with performance on the most commonly utilised interoceptive accuracy measure [37], the Heartbeat Counting Task (HCT) [38], in which participants are asked to count the heartbeats they perceive internally in a given time period (without using external cues such as taking their pulse), and estimates are compared to the objective number of heartbeats that occurred. While empirical findings have been inconsistent, with some studies finding no differences in interoceptive accuracy between those with and without EDs (e.g. [39]), much evidence indicates atypical interoception in those with EDs, with over 90% of studies on EDs finding impairments in interoception relation to control participants, and these difficulties being observed across a range of EDs (AN, BN and BED) and recovered individuals (see [40] for a review). Individuals with, at risk of, or recovered from AN, BN and BED often self-report interoceptive difficulties, both in the domains of accuracy and attention [39, 41–45]. Where objective measures of interoceptive accuracy have been utilized, cardiac interoceptive accuracy has been investigated in those with AN and in those recovered from BN, with both groups exhibiting lower accuracy than typical control participants [46, 47]. Evidence also suggests elevated pain thresholds in individuals with AN and BN [48–50], atypicalities in a range of gastrointestinal interoceptive measures across EDs, including greater gastric capacity and reduced sensitivity to gastric distention in BN [26, 51–53], and atypical taste perception in EDs, such as reduced taste sensitivity in AN, higher pleasantness or intensity ratings of tastes in BN than control samples [54–56]. Atypical interoception may contribute to ED symptomatology, e.g. through reduced recognition of hunger and satiety [47], and reduced perception of the effects of malnutrition, such as fatigue and altered body temperature [39] (see [25] for a review). Individuals with EDs may also attempt to suppress or avoid attending to signals relating to hunger and satiety, potentially leading to reduced interoceptive attention or accuracy [25]. The nature of the relationship between interoception and EDs is therefore likely to be complicated (with interoceptive accuracy and attention both potentially involved), and may be bidirectional. The relevance of interoception to eating disorder pathology is clear, however, with suggestions that interventions to alter interoceptive accuracy or attention are incorporated in ED treatment becoming more prevalent [57, 58].

Interoceptive signals have long been understood to contribute to emotional experience with internal signals featuring prominently in all modern theories of emotion [59, 60]; emotions are thought to emerge when interoceptive signals are interpreted in combination with contextual cues. It is therefore likely that impaired interoceptive accuracy or attention gives rise to some of the emotional difficulties (e.g. difficulties identifying and regulating one’s own emotions and difficulties recognising others’ emotions) commonly experienced by those with EDs [27, 61, 62]. Indeed, alexithymia (difficulties identifying and describing one’s emotions) [63], is common in EDs [64–67], and has been associated with atypical interoceptive accuracy, such as reduced performance on tasks assessing objective interoceptive accuracy (in the cardiac, muscular effort and taste domains) [2, 68, 69] and self-report measures [70, 71]. Atypical interoceptive attention has also been observed in alexithymia, although the direction of this effect varies across studies, likely due to self-report measures being interpreted inconsistently, with some participants reporting on their interoceptive accuracy and others on their interoceptive attention [72]. Alexithymia has also been associated with atypical structure and function of the cortical structures known to support interoception, such as the anterior insula and anterior cingulate cortices e.g. [73–77]. Interestingly, this relationship between alexithymia and interoception has been observed in multiple clinical groups [6, 70], including those with EDs [41, 70]. While some individuals may experience alexithymia owing to non-interoceptive difficulties (e.g. language impairments), interoceptive impairment (potentially affecting both accuracy and attention) is thought to be a common cause of alexithymia (see [78] for a review). Indeed, the original definition of alexithymia describes this construct in terms of reduced accuracy distinguishing between internal signals of emotion, and differentiating emotions from other somatic sensations (i.e. interoceptive accuracy within the emotional domain) [63].

Alexithymia is of relevance to the current study, investigating recognition of others’ interoceptive states, not only because of its strong link with one’s own interoceptive abilities, but also as it is strongly associated with recognition of others’ *emotional* states [79]. Indeed, alexithymia appears to explain difficulties recognising others’ emotions in a number of clinical conditions, such as autism [80, 81] and EDs [27, 67, 82–84]. It is therefore likely that a similar relationship exists beyond the affective domain; difficulties perceiving or identifying one’s own *non-emotional* interoceptive states may be associated with difficulties recognising these interoceptive states in others. While higher interoceptive accuracy appears to predict better recognition of others’ *emotions* [85, 86], research into the ability to recognize other’s non-emotional interoceptive states is lacking. Some studies have investigated pain recognition, and the ability to recognize others’ pain has been associated with alexithymia [83]. Typical participants also appear able to detect illness in others (whether individuals who had received either a bacterial injection triggering an immune response or a placebo injection were ‘sick’ or ‘healthy’) above chance [87]. Another study found that individuals performed above chance at identifying which of two individuals a visual representation of a heartbeat belonged to [88], and some studies have investigated recognition of babies’ cries, for example rating cries in terms of infants’ pain or sickness [89–91]. Beyond these studies, however, there has been no direct investigation of the ability to recognize others’ interoceptive states, beyond emotions.

Accurate recognition of others’ internal states is crucial for successful social interactions; identifying others’ interoceptive states such as hunger, fatigue, pain, and nausea allows one to empathize, respond appropriately, and provide assistance where required. More accurate recognition of others’ interoceptive states should therefore lead to more successful social interactions and relationships, and better ability to provide care for others when needed. This may be particularly relevant to caring roles, such as when parenting or in medical or care-providing professions, and when the observed individual struggles to recognize or communicate their own internal states, as may be the case in children, older adults, and some clinical groups [92, 93]. This is also especially relevant to populations where both atypical interoceptive accuracy/attention and difficulties with interpersonal relationships have been observed, as is the case for many clinical groups, including those with EDs [94–97]. The lack of research on recognition of others’ interoceptive states is therefore surprising, but may be attributable to the lack of available stimuli until now.

The current study aimed to investigate the ability to recognise others’ interoceptive states, and whether this is predicted by participants’ ability to recognise their own internal signals (both emotions and non-emotional interoceptive states). This question was investigated in a sample of typical individuals, and those with EDs, who are likely to experience interoceptive difficulties. It was hypothesized that one’s own interoceptive abilities (assessed using HCT and the Multidimensional Assessment of Interoceptive Awareness) and alexithymia (assessed using the Toronto Alexithymia Scale) would predict one’s ability to recognize others’ interoceptive states. It was also predicted that, before controlling for interoception and alexithymia, the ED group would exhibit impaired recognition of others’ interoceptive states.

## Method

### Participants

Participants were 108 females 40 diagnosed with EDs by an independent clinician, 68 with no ED history). Participants were recruited through adverts in the community (including on social media), university participant pools, ED clinics, the Beat ED charity, and a database of participants who had taken part in previous research on EDs and indicated that they would like to participate in future studies. ED participants completed the Feeding and Eating Disorders section of the SCID-5-RV interview [98], administered by a member of the research team, to confirm current ED status. The ED group was heterogeneous (31 AN, 4 BN, 2 BED, 1 purging disorder, 2 with ED diagnoses unconfirmed by SCID), with illness duration ranging between 1 and 25 years. Given this heterogeneity, analyses were conducted with the entire diagnosed group, as well as in the 31 participants with confirmed AN diagnoses alone. The ED and control groups did not differ significantly in IQ, measured by the two subscale version of the Wechsler Abbreviated Scale of Intelligence [99], meaning any group differences on the behavioural tasks would be unlikely to be driven by differences between the samples in terms of cognitive ability. The ED group was significantly older than the control group, so although the difference in means was relatively small (ED *M* = 29.48, *SD* = 9.35; control *M* = 24.19, *SD* = 8.85), age was controlled for statistically in analyses comparing the ED and control groups, as performance on behavioural tasks, and alexithymia and interoception, may change with age [100]. Eating disorder symptomatology (assessed using the Eating Disorder Examination Questionnaire; EDE-Q) [101]), depression, anxiety and stress, (assessed with the Depression, Anxiety and Stress Scale; DASS [102]) and BMI, were all significantly higher in the ED than control group as expected. See Table 1 for these group comparisons. Participants gave informed consent to participate.

**Table 1:** *t*-tests comparing ED and control groups on demographic variables

Measure	ED Mean (SD)	Control Mean (SD)	*t*	*p*
IQ	105.15 (11.92)	101.07 (11.18)	1.79	.077
Age	29.48 (9.35)	24.19 (8.85)	2.93	.004
ED symptomatology	3.82 (1.27)	1.59 (1.23)	8.98	<.001
Depression	16.87 (12.37)	8.12 (8.89)	3.84	<.001
Anxiety	13.26 (9.37)	7.03 (6.26)	3.67	.001
Stress	21.87 (15.60)	11.85 (8.07)	3.69	.001
BMI	19.19 (7.55)	22.47 (3.70)	2.46	.018

### Interoception

#### Objective interoceptive accuracy

Objective cardiac interoceptive accuracy was assessed using a modified version of the HCT [38]. Participants’ right index finger was placed inside a pulse oximeter while they counted their heartbeats (felt internally, without taking their pulse) across four time intervals (either 25 s, 35 s, 45 s and 100 s or 28 s, 38 s, 48 s and 103 s), presented in a randomized order. Participants’ verbally reported number of heartbeats was compared to the objective number of heartbeats recorded by the pulse oximeter. Participants were instructed not to estimate how many heartbeats occurred [103, 104].

A time estimation task [105] was implemented as a control task [106], in order to reduce the likelihood that HCT performance reflects participants’ ability to estimate time and infer number of heartbeats from their knowledge of their heart rate. Procedure was identical to that for the HCT, except participants estimated seconds rather than heartbeats, and completed the set of intervals not completed in the HCT. Time estimation task and HCT order was counterbalanced.

Accuracy on the HCT was calculated using the formula below, with scores ranging between 0 and 100, and higher scores indicating higher cardiac interoceptive accuracy^2^:

$$\left(\left(1-\left(\textrm{Absolute}\ \left(\left(\textrm{Actual}\ \textrm{number}\ \textrm{of}\ \textrm{heartbeats}-{\textrm{participant}}^{'}\textrm{s}\ \textrm{estimate}\right)/\left(\textrm{Actual}\ \textrm{number}\ \textrm{of}\ \textrm{heartbeats}\right)\right)\ast 100\right)/\textrm{Number}\ \textrm{of}\ \textrm{counting}\ \textrm{periods}\right)\right).$$

#### Self-reported Interoception

Self-reported interoception was measured using the Multidimensional Assessment of Interoceptive Awareness (MAIA) [107], assessing eight facets of interoception across 32 items, namely Noticing (Awareness of uncomfortable, comfortable, and neutral body sensations, e.g. ‘When I am tense I notice where the tension is located in my body’), Not Distracting (Tendency not to ignore or distract oneself from sensations of pain or discomfort, e.g. ‘I distract myself from sensations of discomfort’, reverse scored), Not Worrying (Tendency not to worry or experience emotional distress with sensations of pain or discomfort, e.g. ‘I can notice an unpleasant body sensation without worrying about it’), Attention Regulation (Ability to sustain and control attention to body sensations, e.g. ‘I can pay attention to my breath without being distracted by things happening around me’), Emotional Awareness (Awareness of the connection between body sensations and emotional states, e.g. ‘I notice how my body changes when I feel happy/joyful’), Self Regulation (Ability to regulate distress by attention to body sensations, e.g. ‘I can use my breath to reduce tension’), Body Listening (Active listening to the body for insight, e.g. ‘I listen for information from my body about my emotional state’), and Trusting (Experience of one’s body as safe and trustworthy, e.g. ‘I trust my body sensations’). The MAIA was selected as it includes subscales that explicitly assess interoceptive attention (Attention Regulation and Not Distracting subscales), and has been validated for use in EDs, with findings in an ED sample replicating the original 8 factor structure, and finding acceptable internal and external consistency [41].

#### Alexithymia

At the time of data collection, a well validated self-report measure of interoceptive accuracy was not available. Therefore, alexithymia was measured with the Toronto Alexithymia Scale (TAS-20) [108]. The TAS-20 includes 20 items, assessing three facets (Difficulty identifying feelings, e.g. ‘I am often confused about what emotion I am feeling’, Difficulty describing feelings, e.g. ‘It is difficult for me to find the right words for my feelings’, and Externally oriented thinking, e.g. ‘I prefer to analyze problems rather than just describe them’, reverse scored). TAS-20 scores range between 20 and 80, with higher values indicating more severe alexithymia. The TAS-20 arguably assesses ‘emotional’ interoceptive accuracy, and appears to be strongly negatively associated with objective and more recently developed self-report measures explicitly assessing interoceptive accuracy [70, 71].

### Recognition of others’ interoceptive states

#### Stimuli

**Fig. 1:**
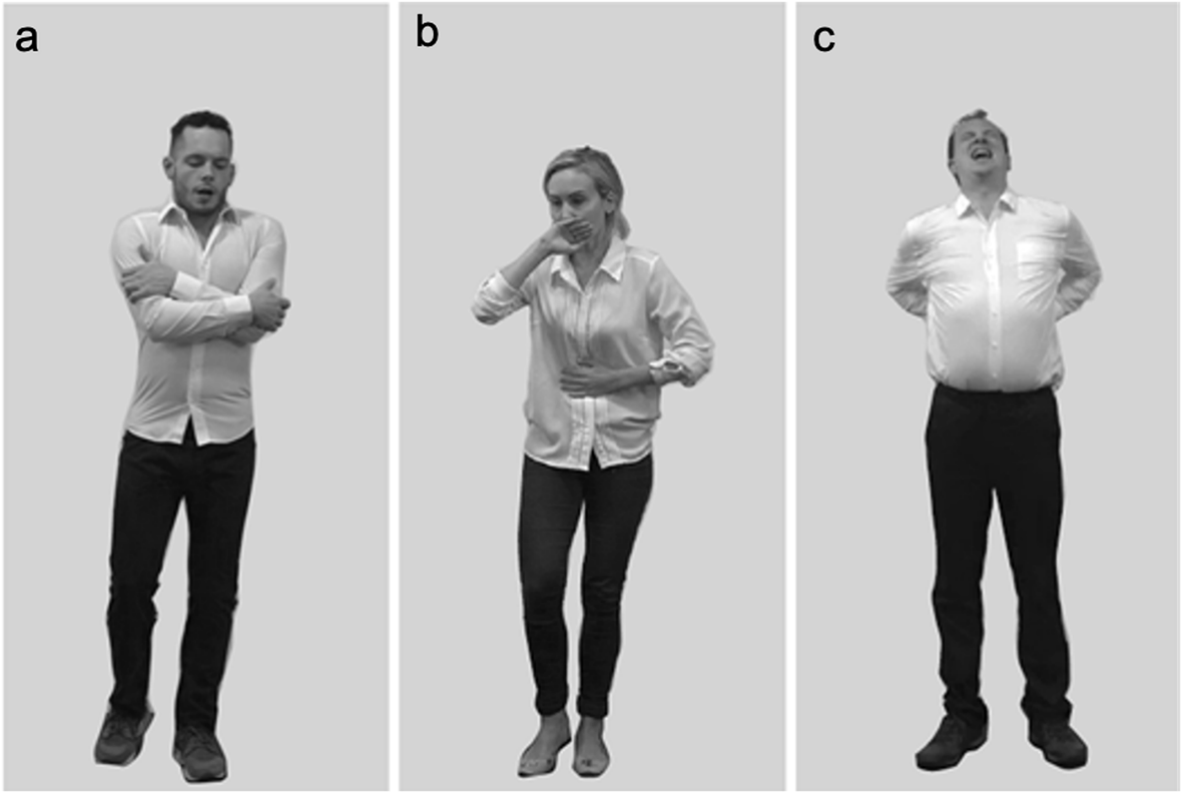
Examples of stimuli expressing non-emotional internal states: Cold (**a**), nausea (**b**) and pain (**c**)

Fifty-six static images depicted four trained actors posing seven interoceptive states (two exemplars of each state per actor): itch, satiety, pain, nausea, cold, breathlessness, and tiredness (Fig. 1). These stimuli were validated prior to use (Supplementary Materials 1).

#### Procedure

The experimental procedure was adapted from that used by Brewer et al. [83] to investigate emotion recognition in EDs. Trials began with a fixation point (1000 ms), followed by an interoceptive state stimulus (800 ms). Participants then indicated whether the image depicted a specific internal state (e.g. ‘Cold: yes or no?’), responding with a key press. Each state was presented 50 times, yielding 350 trials. Stimuli within a state category were selected randomly on each trial.

**Fig. 2:**
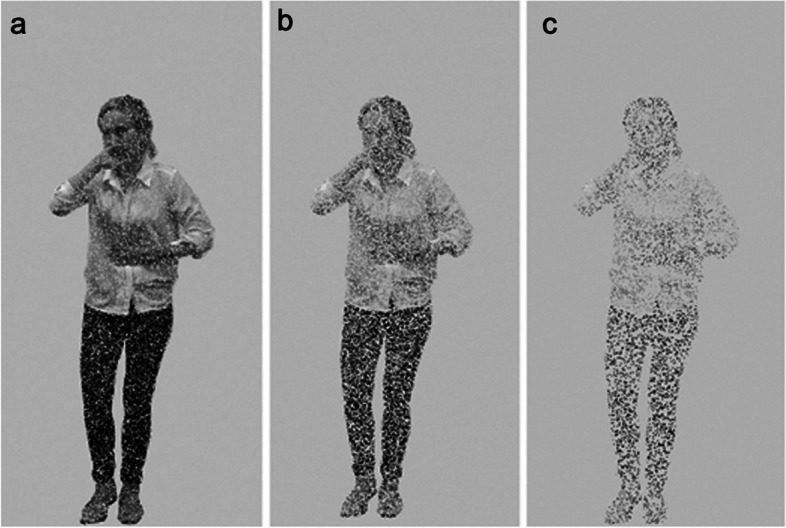
High frequency visual noise obscuring (**a**) 20%, (**b**) 50% and (**c**) 80% of pixels

Participants’ ability to attribute internal states accurately was estimated by their tolerance of high frequency visual noise, (Fig. 2). High frequency visual noise was used to occlude images, in order to avoid ceiling effects common in visual recognition tasks and ensure that the task was sensitive to individual differences in recognition ability. This technique has previously been found to be effective in a similar emotion recognition task in those with EDs [83]. Noise was achieved by replacing the greyscale intensity values comprising the stimulus image with the identical value to the background grey colour. These intensity values were randomly selected across the stimulus image; occluded image points varied across participants, but obscured images uniformly. Visual noise proportion was adjusted using an adaptive staircase procedure. For the first 42 trials (6 trials per internal state), noise level was 50%. For the next 28 trials, noise adjustments were made in 16% increments/decrements according to performance; two consecutive correct responses for the same attribute or one incorrect response lead to increased or decreased noise on the following trial for that attribute, respectively. Subsequently, the noise adjustments were decreased, to 8, 4, 2, and 1%, in blocks of 70 trials. The noise tolerance threshold (% noise) for each attribute at the 350th trial estimated recognition accuracy. The mean noise tolerance threshold across all states was taken as the Global Noise Tolerance Threshold (GNTT). The experimental paradigm was programmed and presented using Matlab with Psychtoolbox [109, 110].

## Results

A Kolmogorov-Smirnov test indicated that noise tolerance threshold was negatively skewed, with five participants (2 ED, 3 typical control) scoring more than twice the interquartile range below the mean. Following removal of these outliers, data were normally distributed, so analyses involving the state recognition task are reported on a final sample of 38 ED and 65 control participants. Given ED heterogeneity, analyses were repeated including only the 31 AN participants, and where this altered the pattern of significance, this is explicitly reported.

Non-normal distributions were also observed for age and a number of questionnaire variables (DASS depression, DASS anxiety, and all MAIA subscales except Not Worrying and Attention Regulation). Noise tolerance thresholds were also not normally distributed for any individual internal state. As LN, log_10_ and square root transformations did not normalise data, untransformed data and non-parametric tests are reported. As the ED and control groups differed in age, age was controlled for in analyses comparing these groups. Where individual participants were missing data (four missing BMI values; three missing HCT values), participants were excluded from analyses including that variable.

### ED and control group comparisons

Independent samples *t*-tests compared the ED and control groups on measures of interoceptive abilities and alexithymia. Higher TAS-20 scores in the ED (*M* = 58.28, *SD* = 11.15) than control (*M* = 52.72, *SD* = 12.93) group, *t* (106) = 2.27, *p* = .025, indicated lower alexithymia in the ED group. This held when age was controlled for in a one-way ANCOVA, *F* [1, 111] = 4.79, *p* = .031, *η*^2^ = .044.

MAIA scores did not differ between the groups for the subscales of Noticing, Worrying, Attention Regulation, Emotional Awareness, or Listening (all *p* > .24), including when controlling for age in one-way ANCOVAs (all *p* > .19). However, the control group scored higher than the ED group on Not Distracting (ED Median = 1.33, N_ED_ = 40; Control Median = 2.67, N_Control_ = 68; *U* = 677.5, *p* < .001), Self-Regulation (ED Median = 1.50, N_ED_ = 40; Control Median = 2.13, N_Control_ = 68; *U* = 996.5, *p* = .020) and Trusting (ED Median = 1.00, N_ED_ = 40; Control Median = 3.00, N_Control_ = 68; *U* = 515.0, *p* < .001) subscales, including when age was controlled for using one-way ANCOVAs (all *p* < .008); the ED group reported being more likely to ignore uncomfortable physical sensations, less able to regulate distress by attending to internal signals, and less likely to experience their body as safe and trustworthy. When the ED group contained only individuals with AN, significantly higher levels of Listening were also reported in the control than AN group (ED Median = 1.00, N_ED_ = 31; Control Median = 1.83, N_Control_ = 68; *U* = 618.0, *p* = .048).

A one-way ANCOVA with HCT performance as the dependent variable, diagnostic group (ED, control) as the independent variable, and age, IQ, BMI and Time Estimation as control variables [104], indicated that the ED and control groups did not differ in cardiac interoceptive accuracy (*F* (1, 96) = .532, *p* = .468, *η*^2^ = .006).

**Fig. 3:**
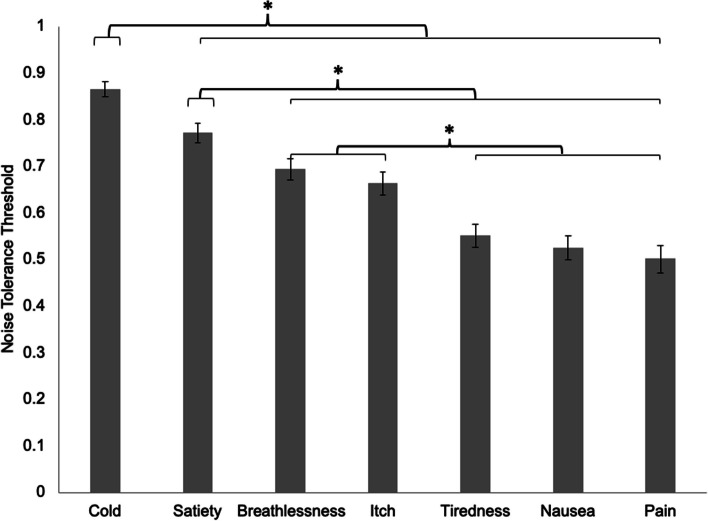
Estimated marginal means for Noise Tolerance Threshold for the seven Interoceptive States. Error bars show standard error. *Significant at α = .05 with Bonferroni correction

A 7(State: Cold, Itch, Nausea, Breathlessness, Satiety, Tiredness, Pain) × 2 (Group: ED, control) ANCOVA controlling for age indicated a significant main effect of State (*F*(6,600) = 4.10, *p* = .001, *η*^2^ = .039), indicating that noise tolerance was higher for some states than others, suggesting better recognition of some states than others. Noise Tolerance was highest for stimuli depicting cold, and lowest for stimuli depicting pain (Fig. 3). A non-significant main effect of Group indicated that noise tolerance threshold did not differ between the ED (*M* = 65.81, *SD* = 12.59) and control (*M* = 65.25, *SD* = 14.08) groups (*F* (1, 100) = .001, *p* = .975, *η*^2^ < .001). The State x Group interaction, *F*(6,600) = 1.13, *p* = .343, *η*^2^ = .011, main effect of age, *F* (1, 100) = .098, *p* = .755, *η*^2^ = .001, and age x State interaction, *F*(6,600) = 1.46, *p* = .196, *η*^2^ = .014, were non-significant.

### Predictors of recognition of others’ internal states

**Fig. 4:**
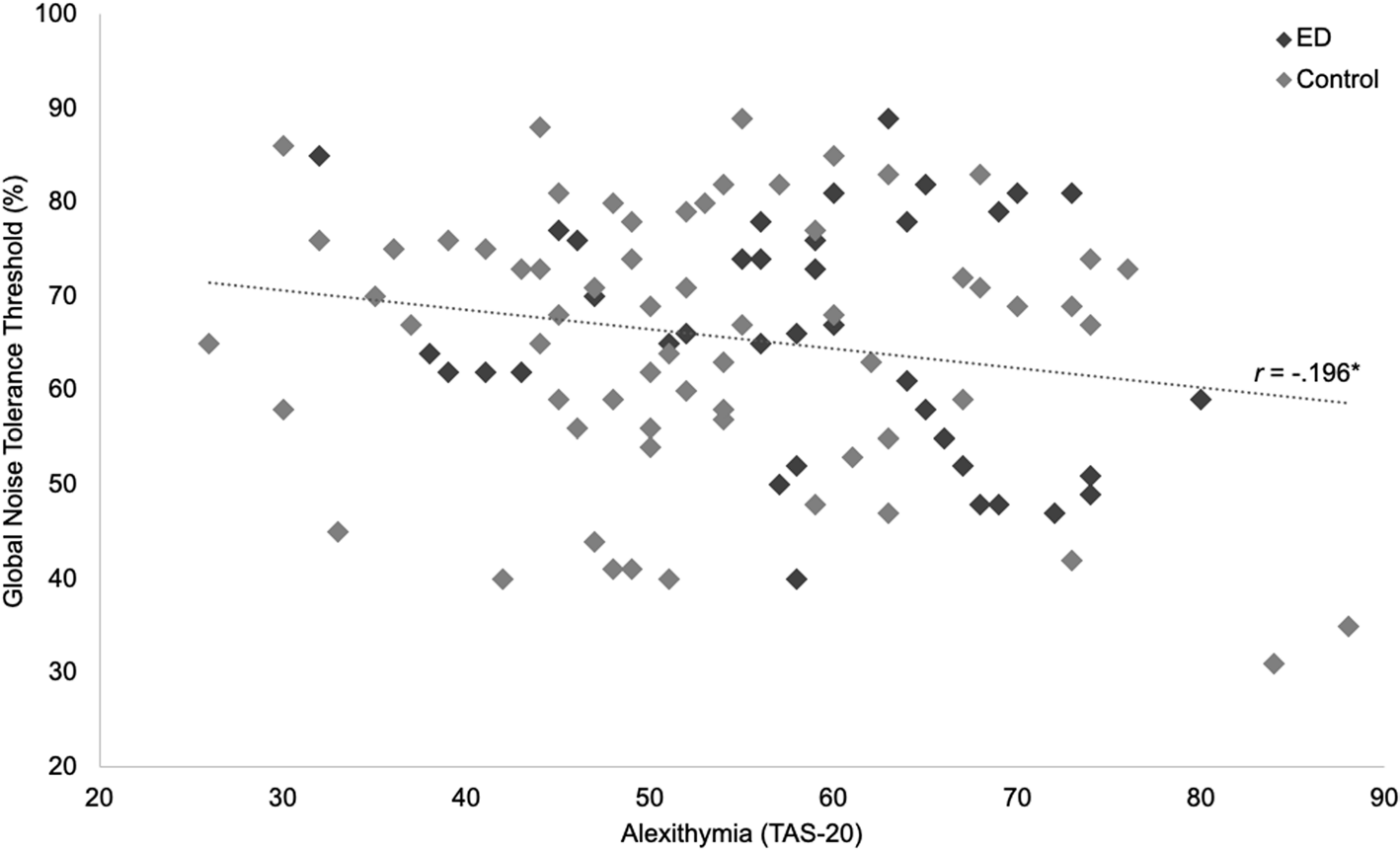
Scatter plot showing significant negative correlation between Alexithymia (measured by TAS-20 total score) and recognition of others’ internal states (measured by mean Noise Tolerance Threshold across all state stimuli

Correlation analyses investigated the relationship between one’s own interoceptive abilities (MAIA subscales, HCT) and alexithymia (TAS-20) and recognition of others’ interoceptive states.^3^ Global Noise Tolerance Threshold (GNTT; mean of the Noise Tolerance Thresholds for all individual states) was significantly negatively correlated with TAS-20 score, *r* (101) = − .196, *p* = .047, indicating poorer recognition of others’ internal states in those with greater difficulties identifying and describing their own emotional internal states (Fig. 4). This fell to a trend when individuals with EDs other than AN were excluded, likely due to reduction in power, *r* (92) = − .191, *p* = .065. Similarly, this effect fell below the significance threshold when the ED, *r* = −.250, *p* = .130, and control, *r* = −.186, *p* = .139, samples were analysed separately, again likely owing to reduced statistical power. EDE-Q scores were not significantly correlated with GNTT, *r* (101) = − .04, *p* = .657, indicating that ED symptomatology was unrelated to recognition of others’ interoceptive states. No significant relationship was observed between GNTT and any MAIA or DASS subscale. Similarly, there was no significant association between GNTT and Heartbeat Counting Task performance in a partial correlation controlling for age, IQ, BMI and Time Estimation, *r* (91) = − .05, *p* = .633. When the AN group only was analyzed, however, both DASS Anxiety, *r* (29) = − .372, *p* = .043, and DASS Depression, *r* (29) = − .485, *p* = .007, were significantly correlated with GNTT, indicating that higher depression and anxiety in those with AN were associated with lower GNTT, suggesting poorer recognition of others’ interoceptive states.

## Discussion

The current study was the first to investigate the ability to recognize others’ interoceptive states, beyond the domain of emotion, and its relationship with one’s own interoceptive abilities and alexithymia. Results indicated that one’s own objective cardiac interoceptive accuracy and self-reported interoceptive attention (estimated by the two MAIA subscales most closely related to the construct of interoceptive attention: the Attention Regulation and Not Distracting subscales) were not related to recognition of others’ interoceptive states. Alexithymia, however, was significantly negatively associated with recognition of others’ interoceptive states, whereby those who struggled to interpret their own emotional internal signals were also less able to recognise visual noise-occluded images of others expressing interoceptive states (such as cold, pain, satiety and itch). ED presence and severity were not related to recognition of others’ interoceptive states, suggesting that it is the ability to recognise one’s own internal states, rather than ED pathology per se, that predicts the ability to identify these states in others.

The negative relationship between alexithymia and recognition of others’ interoceptive states extends previous findings that those with more severe alexithymia struggle to recognize others’ *emotions*, both within the typical population [79], and in clinical groups including EDs [83]. As alexithymia is strongly associated with poor interoceptive accuracy, and many of the items in the TAS-20 refer to ‘sensations in my body’, ‘feelings’, or ‘physical sensations’ rather than ‘emotions’, making them relevant to interoception more broadly, the current findings may be indicative of poor interoceptive accuracy in the self leading to difficulties identifying interoceptive signals in others. The current study may, therefore, have underestimated the relationship between one’s own self-reported interoceptive accuracy and ability to recognize others’ interoceptive states, due to most TAS-20 items relating more specifically to emotion. The recently developed Interoceptive Accuracy Scale (IAS; 64) was explicitly designed to assess subjective interoceptive accuracy outside of the emotional domain, allowing future work to investigate the relationship between self-reported interoceptive accuracy and recognition of others’ interoceptive states more specifically. It is likely that, as the current behavioral task involved recognition of others’ non-emotional interoceptive states, a self-report scale which explicitly refers to perception of these interoceptive states in the self (e.g. the IAS) would be an even stronger predictor of performance than the TAS-20.

The observed relationship between alexithymia and recognition of others’ states has implications for real world social functioning. If difficulties perceiving one’s own emotions and other internal states lead to difficulties recognising interoceptive states in others, this may contribute to social impairment, due to difficulties responding appropriately to others during social interactions. Similarly, these difficulties may impede one’s ability to care for others, which is particularly relevant for individuals with a caregiving role, such as parents, or those working in childcare or medical settings, for example. Indeed, attending to and responding to children’s interoceptive states has been described as an important aspect of parenting, which contributes to the development of empathy and verbal and non-verbal communication skills in children [112]. Further work is required in order to determine the causal nature of this relationship and whether training individuals’ interoceptive accuracy or attention might improve recognition of others’ interoceptive states, and in turn caring and social abilities. Future work should also aim to replicate this finding separately in typical and clinical samples separately, as the current study was underpowered to detect an effect in either sample alone.

Importantly, ED presence and symptom severity themselves were unrelated to recognition of others’ interoceptive states, despite interoceptive atypicalities (in the domain or attention and accuracy) being common in ED [40], and the current ED sample reporting higher alexithymia and lower interoceptive abilities on some MAIA subscales than the control group; those with ED reported being more likely to ignore uncomfortable physical sensations, less able to regulate distress by attending to internal signals, and less likely to experience their body as safe and trustworthy, and those with AN reported low levels of active listening to interoceptive cues for insight into their body state. This is consistent with findings in the emotional domain; while difficulties recognising others’ emotional expressions are common in EDs, these difficulties are associated with co-occurring alexithymia rather than ED symptomatology per se [83]. It is likely that there is substantial variation in the ability to recognize one’s own interoceptive states within the ED population, which may predict individual differences in the recognition of these states in others. Of course, the fact that interoceptive deficits are common in those with EDs may mean that difficulties recognising other’s interoceptive states are more prevalent in this population than in the neurotypical population, but these social perception difficulties are unlikely to be universal in EDs. Notably, when individuals with AN were analyzed separately, anxiety and depression were both negatively associated with the ability to recognize others’ interoceptive states. As both anxiety and depression have been associated with atypical interoceptive accuracy and attention (e.g. Paulus & Stein, 2010), further work with more sensitive measures of interoception is required to determine whether atypical interoceptive accuracy and/or attention mediate this relationship, whether anxiety and depression have a direct and specific influence on recognition of others’ interoceptive states in AN, or whether this effect is simply explained by a more domain-general effect of anxiety and depression on behavioral task performance.

Performance on the HCT was not associated with the ability to recognize others’ internal states, and did not differ between those with and without EDs. As the HCT assesses a single (cardiac) interoceptive signal channel and interoceptive accuracy may not be a unitary construct [111, 113, 114], it is perhaps unsurprising that performance did not predict recognition of others’ interoceptive states outside the cardiac domain, such as satiety, nausea and itch. Future work should therefore investigate objective recognition accuracy of the same interoceptive states in the self and others. This is particularly important given extensive debate in recent years concerning the utility of cardiac perception tasks, in particular the HCT, for example owing to low associations with other tasks assessing cardiac perception [115]. While the need to control for a range of confounding variables [104] has been taken into account in the current study, it remains the case that heart rate knowledge and exteroceptive cues available through the chest wall [116] or pulse oximeter [117] may have contributed to performance. HCT performance should therefore be interpreted with caution, and future work should aim to investigate the relationship with recognition of others’ internal states across a range of objective interoceptive measures.

Recent work has also highlighted the importance of distinguishing between interoceptive accuracy and attention [2, 3]. As a specific self-report measure of interoceptive attention was not available when data collection began, the Attention Regulation and Not Distracting subscales of the MAIA were utilized to assess interoceptive attention, and were not associated with participants’ ability to recognize others’ interoceptive states. Notably, the behavioural task assessed recognition accuracy, rather than attention to others’ states, so it is possible that attention to one’s own interoceptive states relates to attention to others’ interoceptive states, but is less relevant in predicting recognition accuracy. The recently developed Interoceptive Attention Scale [72] will enable further research into the separable roles of one’s own interoceptive accuracy and attention in recognition of others’ internal states, and future work should aim to investigate the relationship between each of these and both attention to and accuracy recognising others’ interoceptive states.

The current study is the first to utilize stimuli depicting a range of interoceptive states in others. The observed correlation between recognition of these images and alexithymia provides support for these stimuli as valid depictions of others’ interoceptive states, and the variability in performance suggests that it is possible to investigate individual differences in recognition ability. This study therefore has important implications for the research field, paving the way towards further investigation of the recognition of others’ interoceptive states, for example across typical and clinical populations, developmentally, at the neural level, and in relation to other areas of cognition. An updated stimulus set adapted from the current stimuli is now freely available for researcher use in addressing such questions [118].

While this study has important implications for the research field and real world, a number of limitations and future directions are worth considering. As discussed, measurement issues have been a key focus of contemporary interoception research, meaning replication using measures explicitly and specifically assessing subjective interoceptive accuracy and attention, and valid objective measures of interoceptive accuracy and attention across a range of interoceptive signal channels is required. Further, the current ED sample included a range of ED diagnoses, and while diagnoses often change over time [29–31], and both interoceptive difficulties [40] and emotion recognition difficulties [67] have been observed across a range of ED diagnostic categories, it is possible that the relationship between recognition of one’s own and others’ non-emotional interoceptive states differs across ED subtypes. Future work should therefore recruit large subsamples in order to compare across groups. Moreover, as interoceptive and social perception difficulties have been observed in multiple clinical groups [6], further work should investigate whether this relationship holds across multiple clinical populations. The current sample also comprised females exclusively, as ED prevalence (at least where AN and BN are concerned) is higher among females than males [119–124], and interoceptive difficulties have also been found to vary as a function of sex, with females reporting greater interoceptive attention, and males often exhibiting higher interoceptive accuracy than females, although this varies with task type [125–127]. Investigating the role of sex in the relationship between recognition of one’s own and others’ interoceptive states, both in the ED and typical population, is a priority, however, especially as recent research suggests that ED psychopathology is more prevalent in males than previously assumed [128, 129]. Finally, while the use of high frequency noise in the current recognition task is likely to have increased the sensitivity of the task to individual differences and avoided ceiling effects, it of course also reduces the ecological validity of stimuli, so replication with more naturalistic tasks is recommended. Replication is also required utilising additional interoceptive states. It is also worth noting that as stimuli could be repeated, the level of noise present during previous presentations may have affected performance on subsequent presentations if individual stimuli were memorised.

## Conclusions

The current study found that alexithymia predicted the ability to recognize others’ interoceptive states. Recognition ability was not predicted, however, by self-reported interoception (including subscales assessing interoceptive attention) or objective cardiac interoceptive accuracy, or by eating disorder presence or symptom severity. Findings suggest that one’s ability to perceive and identify one’s own internal states accurately is associated with the ability to recognize these states in others, with implications for social interactions, relationships, and caring abilities. Future research should replicate these findings using recently developed measures of interoceptive accuracy and attention, across a range of clinical groups.

### Supplementary Information

**Supplementary Material 1.**

## Data Availability

Upon acceptance, all data will be made available through the Open Science Framework (https://osf.io/).

## Abbreviations

EDs: Feeding and eating disorders
AN: Anorexia nervosa
BN: Bulimia nervosa
BED: Binge eating disorder
HCT: Heartbeat counting task
SCID: Structured clinical interview for DSM-5
EDE-Q: Eating disorder examination questionnaire
DASS: Depression, anxiety and stress scale
MAIA: Multidimensional assessment of interoceptive awareness
TAS-20: Toronto alexithymia scale
GNTT: Global noise tolerance threshold

## Footnotes

1 While distinctions between different ED categories have traditionally been made, e.g. based on divergent genetic profiles [28], diagnostic migration is common [29–31] encouraging transdiagnostic approaches that recognize the shared underlying psychopathology of EDs [32–34].

2 Accuracy on the time estimation task was calculated using the same formula, but with number of seconds replacing number of heartbeats.

3 Pearson correlations were used for TAS-20, EDE-Q and MAIA Not Worrying and Attention Regulation subscales, while Spearman correlations were used for DASS anxiety and depression, and all remaining MAIA subscales, due to violated normality assumptions.
