# Supplementary material for: Recognition of others’ interoceptive states in those with and without eating disorders
Source: BMC Psychiatry. 2024 Feb 28;24:169. doi: 10.1186/s12888-024-05615-4 (PMC10900571; doi:10.1186/s12888-024-05615-4)
Supplement: Supplementary file 1 — Supplementary Material 1. [file 12888_2024_5615_MOESM1_ESM.docx]

*Supplementary Materials 1 - Validation of Interoceptive State Stimuli*

Initially, stimuli representing nine internal states (with the addition of hot and hunger) were created through video recordings, and two experimenters selected three static frames that they agreed best represented the nine intended internal states for each actor. One hundred participants viewed these 108 static images and were asked to select which of the nine state options, or an ‘other’ option, best described the internal state experienced. Stimuli depicting ‘hot’ and ‘hunger’ were not recognized above chance or discriminated from other internal states, and were therefore not used in the current study. The seven remaining states were recognized above chance, and were more likely to be labelled as the intended state than any other state (or ‘other’). The two best recognized images for each state for each of the four actors (56 images in total) were selected for use in the current study. See Figure 1 for examples of the interoceptive state stimuli used in the current task.
